# Detection of *Coxiella burnetii* in the mammary gland of a dairy goat

**DOI:** 10.1007/s11259-023-10233-8

**Published:** 2024-01-18

**Authors:** Benjamin Ulrich Bauer, Martin Peters, T. Louise Herms, Martin Runge, Peter Wohlsein, Tim K. Jensen, Martin Ganter

**Affiliations:** 1 University of Veterinary Medicine Hannover, Foundation, Clinic for Swine and Small Ruminants, Bischofsholer Damm 15, Hannover, 30173 Germany; 2 Chemisches und Veterinäruntersuchungsamt Westfalen, Zur Taubeneiche 10-12, Arnsberg, 59821 Germany; 3 Lower Saxony State Office for Consumer Protection and Food Safety (LAVES), Food and Veterinary Institute Braunschweig/Hannover, Eintrachtweg 17, Hannover, 30173 Germany; 4 Department of Pathology, University of Veterinary Medicine Hannover, Foundation, Bünteweg 17, Hannover, 30559 Germany; 5 Center for Diagnostic, Technical University of Denmark, Henrik Dams Allé, Kongens Lyngby, 2800 Denmark; 6 Present Address: Department of Veterinary and Animal Sciences, University of Copenhagen, Grønnegårdsvej 15, Frederiksberg C, 1870 Denmark

**Keywords:** Bulk tank milk, Fluorescence in situ hybridization, Goat, Q fever, Udder, Zoonosis

## Abstract

The zoonotic bacterium *Coxiella (C.) burnetii* can be excreted by infected goats through birth products and milk. The detection of *C. burnetii* DNA in the mammary gland tissue of infected dairy goats and intermittent milk shedders has been reported, but confirmation of *C. burnetii* bacteria in the udder remained pending. The pathogen caused abortions in a 152-head dairy goat herd, resulting in the vaccination against *C. burnetii* of the entire herd with annual boosters. To monitor the *C. burnetii* shedding at herd level, monthly bulk tank milk (BTM) samples were analyzed using PCR (IS*1111*). Despite vaccination, *C. burnetii* DNA was detected in BTM samples within the first 16 months of the study. Therefore, individual milk samples were tested on four different occasions several months apart to identify potential intermittent milk shedders. Only one goat (#67455) tested positive three times. This goat was necropsied to investigate the presence of *C. burnetii* in the udder and other organs. PCR detected *C. burnetii* DNA solely in both mammary glands and the left teat cistern. Immunohistological examination identified *C. burnetii* antigen in mammary gland tissue, confirmed by the detection of *C. burnetii* bacteria in the mammary epithelial cells using fluorescence in situ hybridization. The removal of goat #67455 led to negative BTM samples until the end of the study. The findings demonstrate the occurrence of *C. burnetii* in the mammary gland of a naturally infected and vaccinated goat. The presence possibly contributed to intermittent milk shedding of goat #67455, and the mammary gland tissue may serve as a replicative niche for *C. burnetii*.

## Introduction

Q fever is a worldwide zoonosis caused by the obligate intracellular bacterium *Coxiella (C.) burnetii*. Ruminants are considered the primary reservoir and the pathogen replicates particularly in the trophoblasts of the placenta, with the rate of replication increasing toward parturition (Celina and Cerný 2022; Roest et al. 2012). Up to 10^9^ organisms per gram may be present in one gram of placental tissue (Arricau-Bouvery et al. 2003). Infected animals may suffer from reproductive disorders and excrete the bacteria during abortion or parturition through birth products (placenta, amniotic fluid), but also through milk and feces (Bauer et al. 2020; Ullah et al. 2022). In experimentally infected goats, *C. burnetii* DNA was detected in milk between 38 and 52 days after parturition (Roest et al. 2012). In contrast, bulk tank milk (BTM) from naturally infected dairy goat herds remained *C. burnetii* positive for several months (Álvarez-Alonso et al. 2018; Bauer et al. 2022). Therefore, monitoring dairy goat herds with BTM samples analyzed by ELISA or PCR has been proven to be effective to identify *C. burnetii*-positive herds (Jansen et al. 2021; van den Brom et al. 2012; Vellema et al. 2021). Both methods can detect a within-herd prevalence of at least 15% (van den Brom et al. 2012). Given that these methods operate on distinct principles, the concordance between the two assays is satisfactory, despite the ELISA showing reduced sensitivity and specificity compared to the PCR (van den Brom et al. 2012). Intermittently *C. burnetii* shedding goats resulted in discontinuously positive BTM samples, even though the animals were vaccinated (Boarbi et al. 2014; van den Brom et al. 2013). Therefore, it is important to identify intermittent shedders to reduce the risk of transmission. Although, the route of transmission through milk is still not fully understood but experimental studies in the past have shown successful *C. burnetii* infection through the teat canal (Williams 1991). In recent years the pathogen was detected, using PCR methods, in the mammary gland and mammary lymph nodes in goats (Roest et al. 2012; Sánchez et al. 2006; van den Brom et al. 2013). However, the conclusive evidence of the occurrence of *C. burnetii* in the caprine mammary gland tissue through histopathological techniques such as immunohistochemistry and fluorescence in situ hybridization is still pending. The detection of *C. burnetii* DNA in milk and udder tissue from naturally and experimentally infected goats, along with the propagation of *C. burnetii* isolates from goats in bovine mammary gland epithelial cells in vitro, raises the question whether the udder acts as replicative niche for *C. burnetii* (Roest et al. 2012; Sánchez et al. 2006; Sobotta et al. 2022; van den Brom et al. 2013).

The world’s largest human Q fever outbreak was recorded in The Netherlands and was associated with large dairy goat farms in the south of the country. From 2007 to 2011, more than 4,000 people contracted Q fever and the number of infected people is estimated to be about 40,000 (Reedijk et al. 2013; van Roeden et al. 2018). The main route of infection for humans and animals is inhalation of contaminated aerosols. In humans, approximately 40% of infected individuals show flu-like symptoms such as fever, headache, myalgia and pneumonia (Eldin et al. 2017). In the long term, up to 20% of patients with acute Q fever develop chronic fatigue syndrome (Morroy et al. 2016), and affected patients with cardiovascular lesions suffer from endocarditis and vascular disease (Eldin et al. 2017). The clinical impact of consuming raw milk and raw milk products contaminated with *C. burnetii* is still under debate (Barandika et al. 2019; Pexara et al. 2018). A study of prison inmates demonstrated that consumption of raw milk contaminated with *C. burnetii* resulted in seroconversion but not the development of clinical symptoms (Benson et al. 1963). Macrophages are the primary target cells of *C. burnetii*, and it is assumed that the higher numbers in the lungs compared to the gastrointestinal tract makes infection with *C. burnetii* by inhalation more effective than oral ingestion of the pathogen (Gale et al. 2015). Nevertheless, regular consumption of raw milk containing *C. burnetii* has resulted in Q fever symptoms in a few cases (Fishbein and Raoult 1992; Signs et al. 2012). Dupont and colleagues (1992) even suggest a link between raw milk consumption and *C. burnetii*-associated hepatitis. Currently, the risk of contracting Q fever from consuming raw milk and raw milk products is considered to be low but not negligible (Gale et al. 2015; Pexara et al. 2018). Due to the increasing popularity of raw milk consumption and new ways of selling raw milk through vending machines and internet sales (EFSA Panel on Biological Hazards 2015), there is a need to raise the awareness of food-borne pathogens in these products. The process of high-temperature short-time pasteurization inactivates *C. burnetii* in milk (Wittwer et al. 2022).

For the prevention and control of Q fever in goat herds, an inactivated *C. burnetii* Phase I vaccine (Coxevac^®^, Ceva Santé Animale, Libourne, France) is licensed in several European countries for cattle, goats, and recently for sheep. *C. burnetii* shedding is greatly reduced, but not completely prevented, when goats are vaccinated before infection occurs (Arricau-Bouvery et al. 2005). The vaccine is also unable to prevent excretion both during a Q fever outbreak and in the subsequent kidding season (Bauer et al. 2020, 2022; De Cremoux et al. 2012).

The primary objective of the study was to monitor the shedding of *C. burnetii* at the herd level using BTM samples from a highly infected dairy goat herd that was subsequently vaccinated. Despite applying a strict vaccination protocol, the BTM samples continued to test positive for *C. burnetii* DNA using PCR. Therefore, the second study objective was to identify potential intermittent milk shedders by analyzing individual milk samples and to detect the presence of *C. burnetii* bacteria in the mammary gland tissue.

## Material & methods

### Herd history

A dairy goat herd with 152 goats in the German federal state of North Rhine-Westphalia reported abortions, stillbirths and weak kids at the end of the kidding season in April 2018. Four aborted fetuses with placentas from two goats were examined by the North Rhine-Westphalia state laboratory to determine the cause of abortion. The samples were tested for the presence of *Brucella* spp, *Campylobacter* spp., *Chlamydia* spp., *C. burnetii*, bluetongue virus, pestivirus and Schmallenberg virus. The only abortifacient pathogen detected was *C. burnetii* (Cq 11–22, VetMAX™ *C. burnetii* Absolute Quant Kit, Thermo Fisher Scientific GmbH, Dreieich, Germany). The *C. burnetii*-DNA obtained from the placenta was further analyzed with MLVA/VNTR genotyping as previously described (Frangoulidis et al. 2014), and genotype associated with the German sheep population, A3, was identified. The farmer asked the Clinic for Swine and Small Ruminants at the University of Veterinary Medicine Hannover, Foundation, Hannover, Germany, for help in controlling the Q fever outbreak in her dairy goat herd. More details about the Q fever outbreak have been published elsewhere (Bauer et al. 2022).

### Vaccination

Shortly after the detection of *C. burnetii* in April 2018, all adult goats were vaccinated twice at three-week intervals with an inactivated *C. burnetii* Phase I vaccine (Coxevac^®^, Ceva, Libourne, France) according to the manufacturer’s instructions. A booster vaccination was given to all adult goats in July 2019 and July 2020, and the female progeny received their primary vaccination (as described above) four weeks before the start of the breeding season.

### Monitoring

To monitor *C. burnetii* excretion in milk at herd level, monthly BTM samples were collected from April 2018 until December 2021, with one sample missing in September 2020. Due to the irregular detection of *C. burnetii* DNA in the BTM, individual milk samples were taken from the continuously milked goats (n = 96) in January 2020 and colostrum samples (< 24 h post-partum) were collected from freshly kidded goats (n = 69) during kidding season 2020 (March-April 2020). This sampling period is defined as “Spring Sampling 2020”. Individual milk samples were taken again from all lactating goats in July 2020 (n = 162) and November 2020 (n = 150). Pregnant goats were dried off six weeks before the start of kidding in 2021. Again, all continuously milked goats were sampled by individual milk samples in March 2021 (n = 93) and colostrum was taken from freshly kidded goats (n = 49) during kidding season 2021 (March-April 2021). The sample collection in March 2021 and during kidding season 2021 represent the “Spring Sampling 2021”. The number of goats sampled varied on each sampling date due to animal losses or sales. The colostrum and milk samples were collected from both mammary glands from the same goat, under aseptic conditions, in one milk tube and stored at -20 °C until further analysis.

### Dairy goat #67455

*C. burnetii* DNA was detected in individual milk samples from dairy goat #67455(born January 2013) on three of the four sampling dates. This goat gave birth to healthy triplets on 26^th^ February 2018 and was milked continuously until removed from the herd. The goat received its primary vaccination in 2018 and was boostered twice in July 2019 and 2020. Only this goat was included in further examinations. From 20^th^ May to 16^th^ June 2021 (28-day period), individual milk samples from goat #67455 were collected separately from both mammary glands before the start of morning milking and under aseptic conditions. The specimens were stored at -20 °C until further PCR analysis. Moreover, sterile milk samples (sterile plastic tubes with boric acid, KABE-Labortechnik GmbH, Nümbrecht, Germany) were taken from each mammary gland to determine somatic cell counts (SCC) and microbiological composition before the goat was euthanized. In addition, a serum sample was tested for antibodies against caprine arthritis encephalitis virus (CAEV) using a commercial ELISA according to the manufacturer’s recommendations (IDVet, Grabels, France) and gave a negative result. The goat was euthanized with an intravenous injection of sodium pentobarbital (Euthadorm^®^, CP-Pharma GmbH, Burgdorf, Germany) and the necropsy was performed on the same day at the North Rhine-Westphalia state laboratory. A complete necropsy was performed, strictly avoiding contamination with milk. The udder was cut open last, taking strict care not to mix the milk from both halves of the udder. Tissue samples were taken from both lactiferous glands. One specimen was collected bilaterally from each udder, including lymph nodes, mammary gland tissue, lactiferous ducts, gland cistern, teat cistern, and teat canal. Additionally, tissue samples were taken from the reproductive tract (ovaries, oviducts, uterine horns, uterus, and lymph nodes). A new sterile biopsy punch (Ø 8 mm, Kruuse, Langeskov, Denmark) was used for each tissue sample from both lactiferous glands and the reproductive tract. The biopsy punches were transferred to plastic tubes with screw caps before molecular analysis to prevent cross-contamination with *C. burnetii*. Swab samples from the uterus and vagina were collected. Specimens from the hematopoietic system (spleen, thymus), liver, urinary tract (renal pelvis, urine), respiratory tract (lymph nodes, lungs), cerebrospinal fluid and feces were also included in the examinations (Table 1). The non-tissue samples and one sample of each tissue specimen were stored at -20 °C for PCR analysis, and a second tissue sample was fixed in 10% phosphate buffered formalin for histopathology.

**Table 1:** Overview about collected tissue and non-tissue samples from goat #67455 during necropsy. Samples were analyzed by real-time PCR (IS*1111*). Paired organs were always sampled bilaterally. The results refer to both sides unless there are special indications

Tissue sample	PCR result (Cq value)
**Udder**	
Supramammary lymph nodes (*Lymphonodi mammarius)*	neg.
Mammary gland tissues (*Lobi glandulae mammariae*)^a^	left: Cq 33.8; 34.3; neg. right: Cq 37.7; 37.1; neg.
Lactiferous ducts (*Ductus lactiferi*)	neg.
Gland cisterns (*Pars glandularis sinus lactiferi*)	neg.
Teat cisterns (*Pars papillaris*)	left: Cq 37.9 right: neg.
Teat canals (*Ductus papillaris*)	neg.
**Reproductive System**	
Ovaries	neg.
Oviducts	neg.
Uterine horns	neg.
Internal iliac lymph nodes (*Lymphonodi iliaci interni*)	neg.
Uterus	neg.
**Others**	
Liver	neg.
Spleen	neg.
Lungs	neg.
Lung lymph nodes	neg.
Thymus	neg.
Renal pelvis	neg.
Urinary bladder	neg.
**Non-tissue samples**	
Uterus swab	neg.
Vaginal swab	neg.
Urine (bladder)	neg.
Cerebrospinal fluid	neg.
Feces (rectum)	inhibited

^a^three samples each side

### Laboratory examination

#### Molecular analysis of milk and tissue samples

The BTM samples were prepared for PCR analysis as follows:

2 mL of the BTM was centrifuged for 5 min at 2655 g. Subsequently, the fat was removed from the tube with a sterile swab. After another centrifugation step for 10 min at 20,817 g, the supernatant was disposed of. Bacterial DNA was prepared from the remaining pellet using the InviMag^®^ Universal Kit/ KF96 (STRATEC Molecular GmbH, Berlin, Germany) according to the manufacturer’s instructions.

In the case of tissue samples, 20 mg of each sample was mixed with 400 µL molecular biology grade water and then crushed with a steel bullet using the TissueLyser^®^ (QIAGEN, Venlo, Netherlands) for 2 min at 15 Hz. Afterwards 200 µL were put in the InviMag^®^ Universal Kit/ KF96 (STRATEC Molecular GmbH, Berlin, Germany) and the bacterial DNA was prepared according to the manufacturer’s instructions.

The extracted DNA from the BTM samples were analyzed with a commercially available real-time PCR (LSI VetMAX™ *C. burnetii* Absolute Quant Kit, Thermo Fisher Scientific GmbH, Dreieich, Germany), which targets IS*1111*. The manufacturer indicates Cq values ≤ 45 as positive. *C. burnetii*-specific DNA fragments in the individual milk, colostrum and organ samples were detected by amplification of the IS*1111* elements with an in-house real-time PCR according to Frangoulidis and colleagues (2012) due to limited resources. Cycle Quantification (Cq) values ≤ 45 were indicated as positive and Cq values > 45 as negative values. The detection limit for both real-time PCRs is 1 genome equivalent (GE) per PCR evaluated with the Nine Mile genome containing 20 copies of IS*1111* per GE.

#### Cytological and microbiological analysis of the milk samples

Both milk samples collected shortly before euthanasia of goat #67455 were sent for routine cytological and bacteriological analysis at a specialized laboratory (MBFG, Wunstorf, Germany), to identify any bacteria which may cause mastitis, which could interfere with the histopathological findings from the udder tissue. In brief, the somatic cell count (SCC) of the milk was determined using a fluorescence-based optical method (Fossomatic™ 360, Type 15,700, Foss, Hilleroed, Denmark), and microbiological analysis was performed by incubating a milk smear on Aesculin Blood Agar (Oxoid Deutschland GmbH, Wesel, Germany) for 48 h at 37 °C under aerobic conditions.

### Histopathology and immunohistochemistry

For histology, several tissue samples from both mammary glands were examined. Immunohistochemistry (IHC) was performed on both mammary gland tissues and the left teat cistern due to positive PCR results (Table 1). The tissues were fixed, dehydrated, cleared, and processed into paraffin wax blocks. Sections of 3 μm were cut and routinely stained with hematoxylin/eosin and immunostained for the presence of *C. burnetii* antigen. Immunohistochemical staining of *C. burnetii* antigen was performed as described previously (Baumgärtner et al. 1988). Briefly, dewaxed and rehydrated tissue sections were incubated with a polyclonal antibody rabbit antibody specific for *C. burnetii*, followed by a biotinylated goat-anti-rabbit antibody and the avidin biotin peroxidase complex (ABC method Vectastain^®^, Vector Laboratories, Burlingame, CA, USA) according to the instructions of the manufacturer. The immunohistological reaction was visualized using 3,3´-diaminobenzidine-tetrahydrochloride (DAB) as chromogen. Tissues from mice experimentally infected with *C. burnetii* were used as a positive control (Baumgärtner et al. 1988).

### Fluorescent in situ hybridization (FISH)

FISH was performed on 3 µm thick tissue sections from the left and right mammary gland tissue according to Buijs et al. (2022). Briefly, four oligonucleotide RNA-probes (S-S-C.burnetii-188, S-S-C.burnetii-631, S-S-C.burnetii-826, and S-S-C.burnetii-1462) targeting different locations of the 16S ribosomal RNA of *C. burnetii*, were used in a mixture: The oligonucleotide probes were labeled at the 5’ and 3’ end with fluorescein isothiocyanate (FITC) (green) (Eurofins MWG Operon, Ebersberg, Germany). Hybridization was performed at 45 °C with 40 µL of hybridization buffer (100 mM Tris [pH 7.2], 0.9 M NaCl, 0.1% sodium dodecyl sulphate) and 200 ng of each probe for at least 16 h in a Sequenza slide rack (Shandon^TM^, Thermo Fisher Scientific, Rosklide, Denmark). After hybridization, sections were washed three times with hybridization buffer at 45 °C for 15 min and subsequently three times with washing buffer (100 mM Tris [pH 7.2], 0.9 M NaCl). Sections were rinsed in water, air dried, and mounted in Vectashield (Vector Laboratories, Newark, CA, United States) for fluorescence microscopy. An Axioplan2 epifluorescence microscope (Carl Zeiss, Oberkochen, Germany) equipped with a 100-W HBO lamp and filter sets 24, 38 and 43 was used to examine the hybridized specimens. Positive identification of *C. burnetii* was based on a specific hybridization signal from coccoid intracytoplasmic organisms. Images were obtained using an AxioCam MRm version 3 FireWire monochrome camera and the AxioVision software, version 4.5 (Carl Zeiss, Oberkochen, Germany). Tissue sections from a case of spontaneous bovine placenta infection with *C. burnetii* were used as a positive control (Wolf-Jäckel et al. 2021).

### Statistical analysis

The PCR results of the daily collected milk samples (20^th^ May to 16^th^ June 2021) from goat #67455 were analyzed using a Mann-Whitney U test (GraphPad Prism 9, Cypress, CA, USA). A result of *p* < 0.05 was considered significant.

## Results

### Bulk tank milk samples

**Fig. 1:**
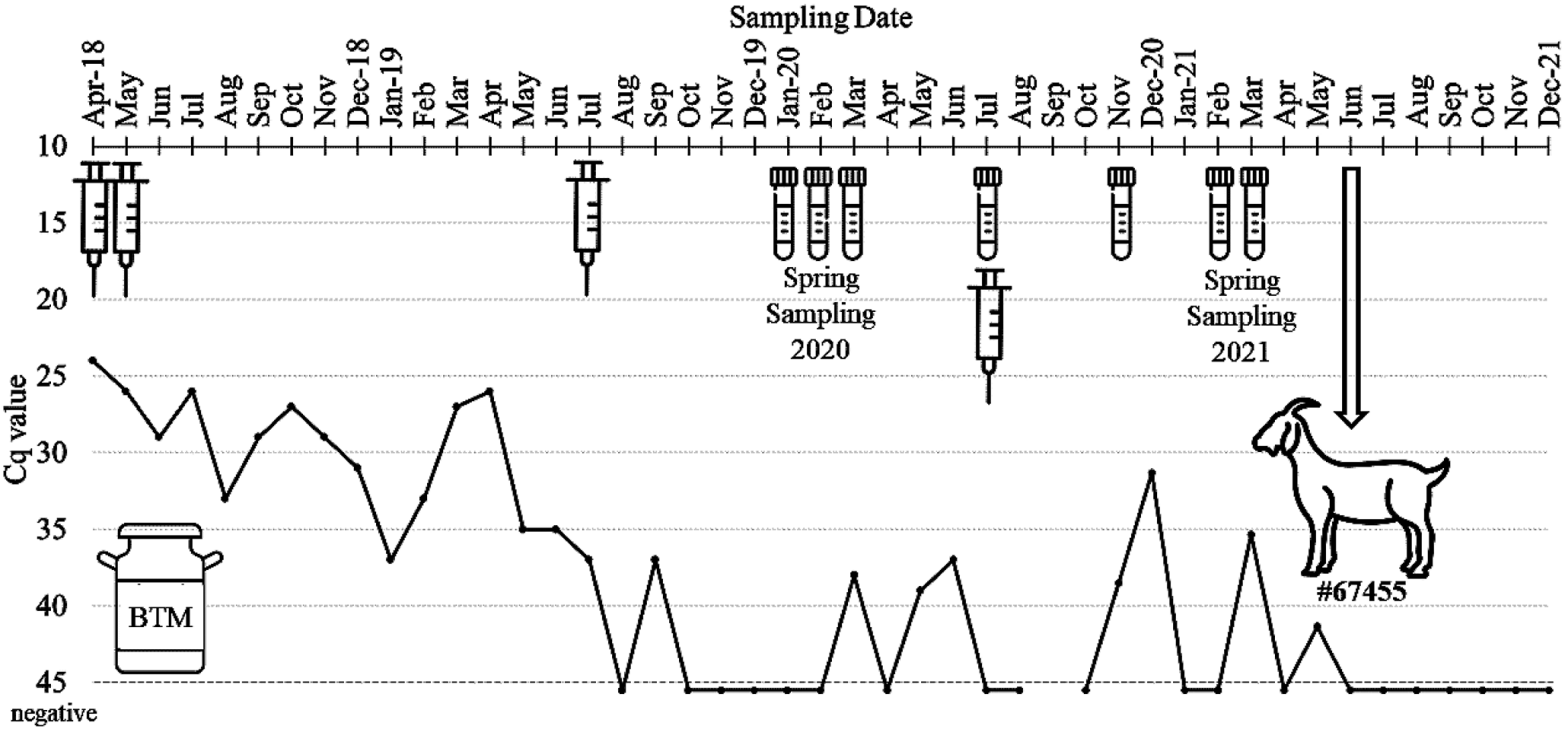
Monthly detection of *C. burnetii* DNA (Cq ≤ 45) in bulk tank milk (BTM) determined by real-time PCR (IS*1111*) from a naturally infected dairy goat herd (April 2018 until December 2021). The syringes symbolize the dates of vaccination against *C. burnetii*. Tubes indicate the sampling of individual milk specimens from goats. In June 2021, goat #67455, which shed *C. burnetii* intermittently, was removed from the herd and was necropsied. BTM in September 2020 is missing

After *C. burnetii* was diagnosed in the dairy goat herd, BTM samples remained positive for 16 months. Thereafter, the results showed an undulating trend with negative outcomes from October 2019 until February 2020. In June 2021, the intermittent shedder (goat #67455) was removed from the herd and subsequent monthly BTM specimens tested negative until the end of the study. The results are shown in detail in Fig. 1.

### Individual milk samples

**Fig. 2:**
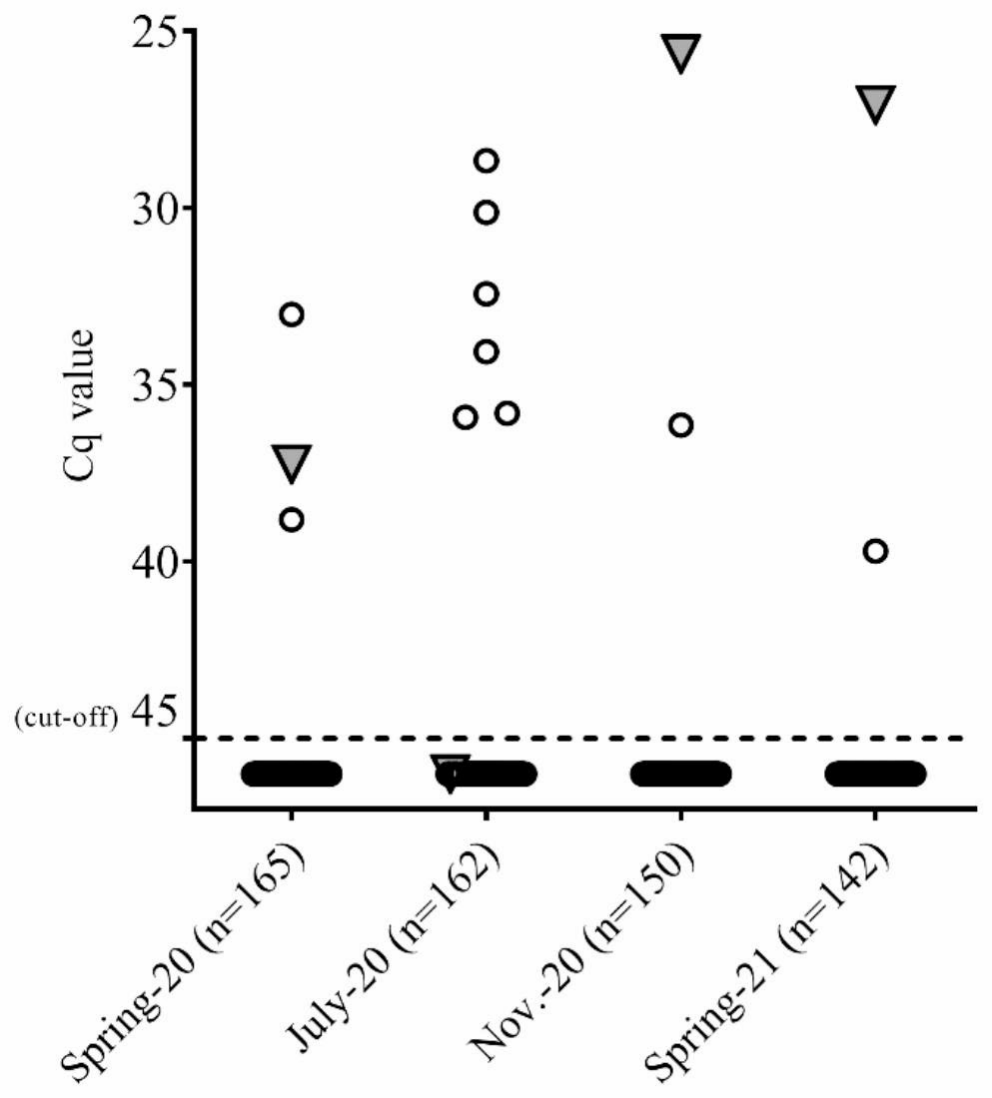
*C. burnetii* DNA in individual milk samples from different sampling periods/dates. Figures in parentheses represent numbers of sampled animals. Only goat #67455 tested positive several times (triangle). All other positive tested goats shed *C. burnetii* once. The dashed line indicates the cut-off value of the PCR assay (Cq ≤ 45)

Eleven goats tested positive for *C. burnetii* in individual milk samples during the four sampling periods/dates. The eleven goats had already been involved in the *C. burnetii* infection in 2018. Of these, ten goats contained *C. burnetii* DNA in their milk only once. Goat #67455 was the only animal that tested positive for *C. burnetii* on three of the four sampling dates. This goat was continuously milked and no colostrum specimens were available in the 2020 and 2021 kidding seasons. Details of the detection of *C. burnetii* DNA in individual milk samples are illustrated in Fig. 2.

### Individual milk samples goat #67455

**Fig. 3:**
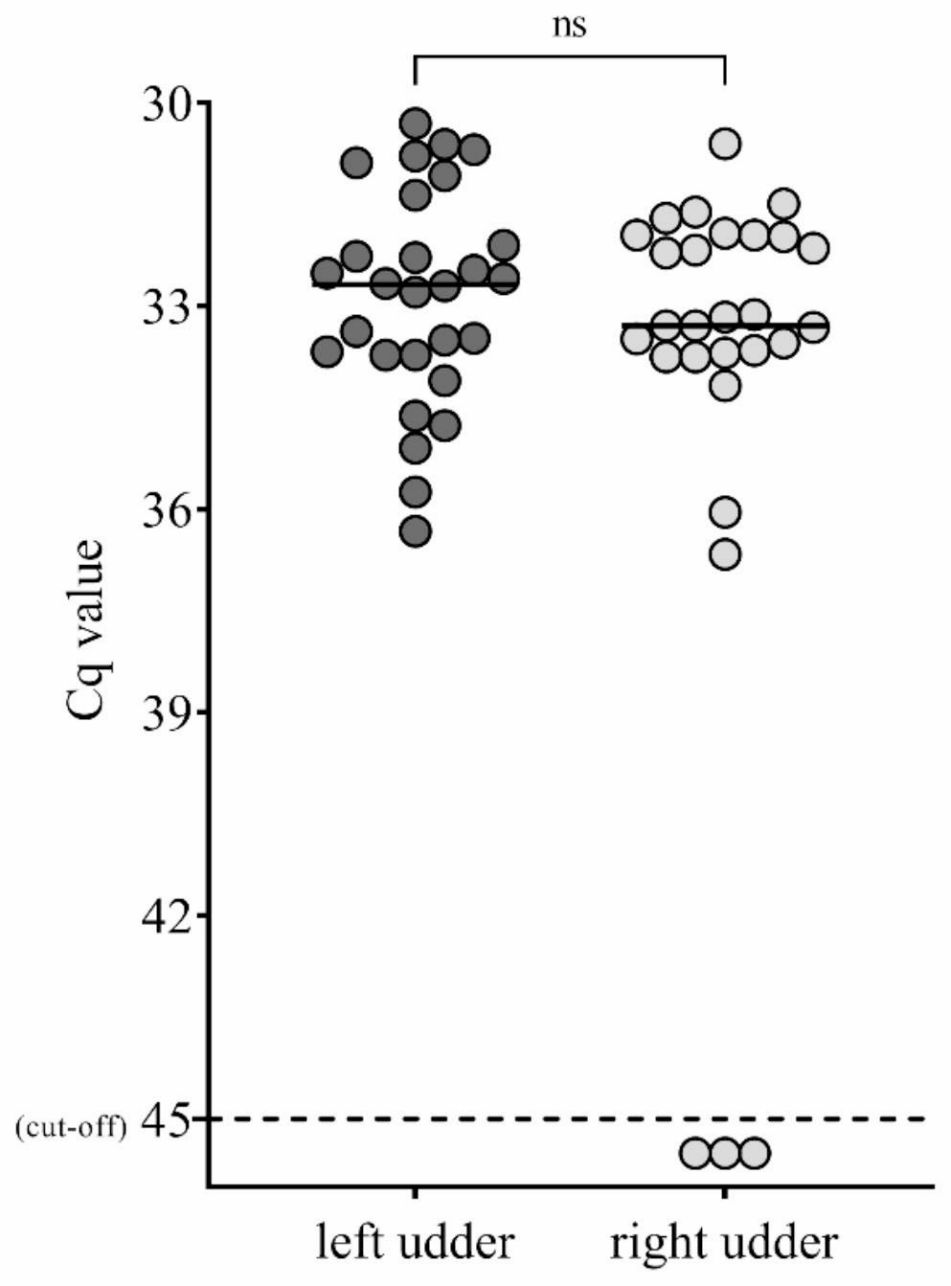
Daily results of *C. burnetii* DNA (Cq ≤ 45) detected in milk samples by real-time PCR (IS*1111*) from both udder halves from goat #67455 during 28-day sampling period. Bars indicate the median Cq value from each udder half. ns = not significant (*p* ≥ 0.05)

During the 28-day study period, all individual milk samples from the left mammary gland of goat #67455 tested positive for *C. burnetii* DNA. Twenty-five samples from the right mammary gland contained *C. burnetii* DNA (Fig. 3). There was no significant difference in the obtained Cq values between the two mammary glands.

### Cytological and microbiological results

The SCC of the left and right udder half from goat #67455 were 299 × 10^3^ cells/ml and 270 × 10^3^ cells/ml, respectively, on the day of euthanasia (22^nd^ June 2021). No pathogens were detected by the cultural bacteriological examination.

### Molecular analysis of tissue samples

DNA of *C. burnetii* was detected in both samples from both mammary tissues and in the left teat cistern. All other tissue samples tested negative by real-time PCR (Table 1).

### Histopathology and immunohistochemistry

**Fig. 4:**
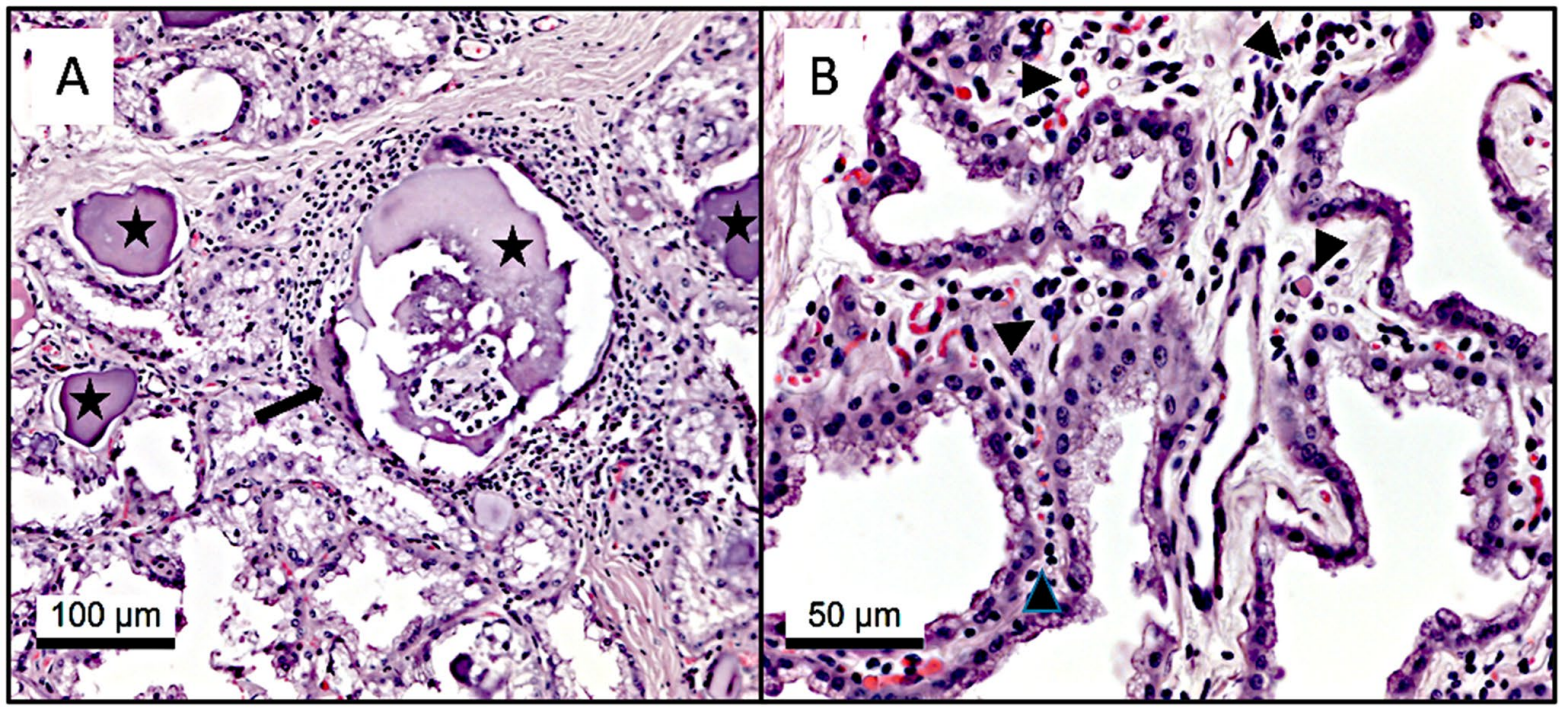
**A**. Lactating mammary gland with interstitial lymphocytic, focally granulomatous inflammation surrounding a corpus amylaceum (asterisks) characterized by macrophages and single multinucleated giant cell (arrow). **B**. Lymphoplasmacytic infiltrates (arrowheads) in the interalveolar interstitium, HE-Staining

Histological examination of the mammary gland tissue of both udder halves revealed multifocal mild, predominantly lymphoplasmacytic interstitial inter-, intralobular and periductual infiltrations (Fig. 4). In alveoli and ducts of the gland, there were several corpora amylacea focally surrounded by multinuclear macrophages.

**Fig. 5:**
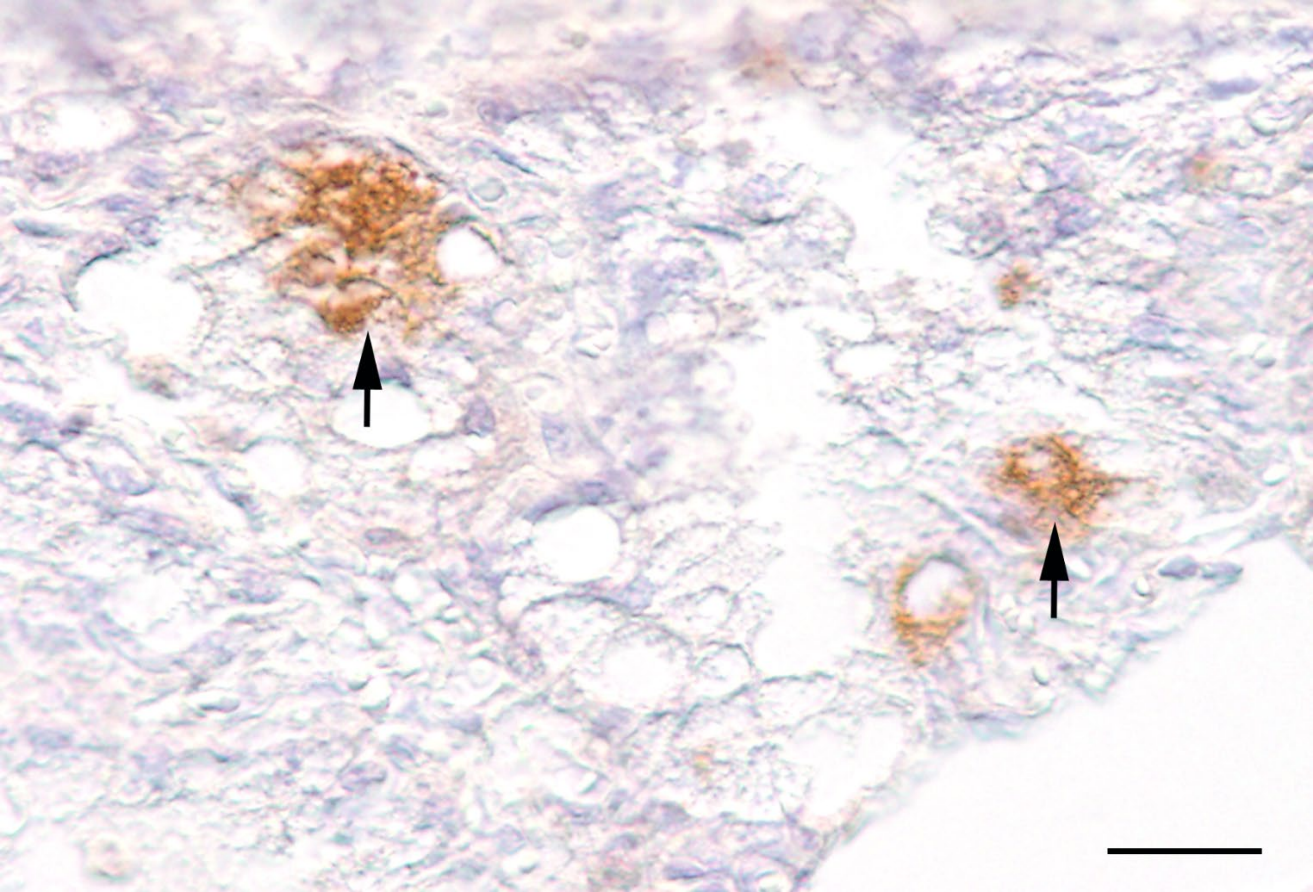
Immunohistochemical labeling of *C. burnetii* antigen in the cytoplasm of single epithelial cells (arrows). Bar = 50 μm

In both mammary gland tissues, single epithelial cells revealed cytoplasmic granular labeling (Fig. 5). The left teat cistern showed no immunolabeling.

### Fluorescent in situ hybridization (FISH)

**Fig. 6:**
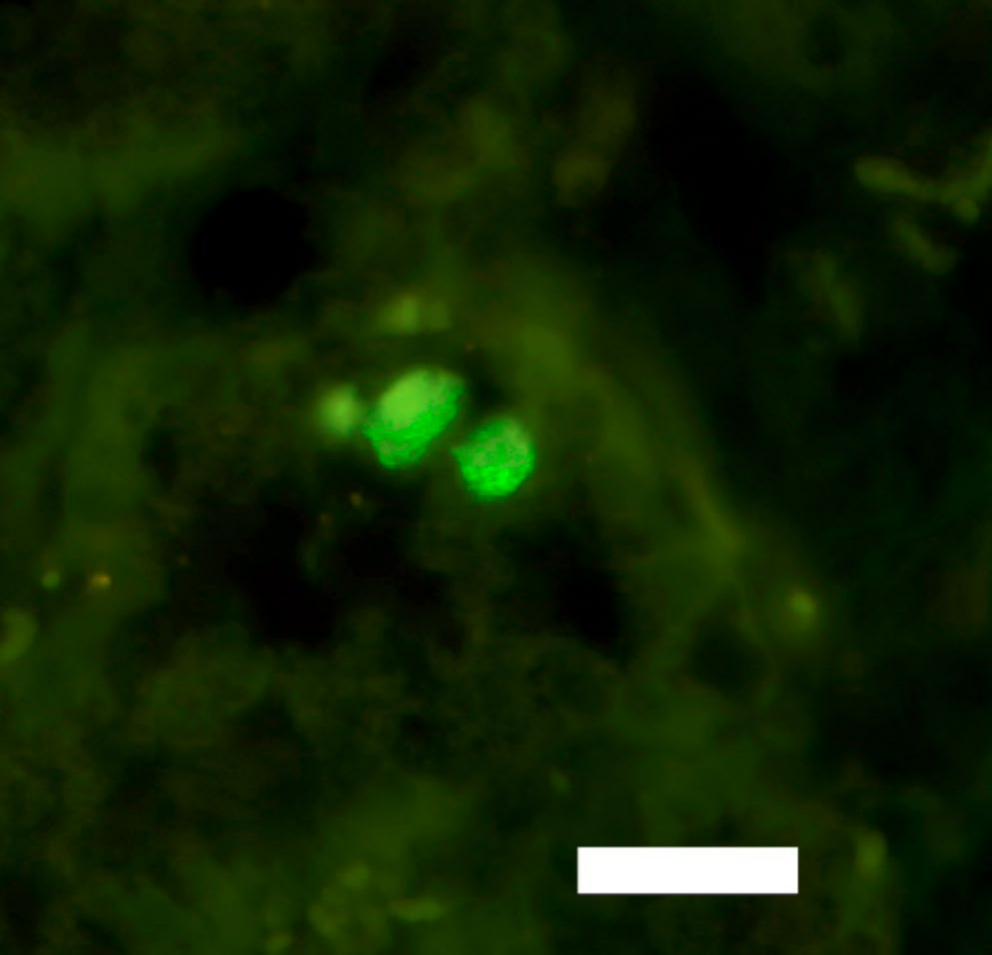
Caprine Mammary gland. Detection of *C. burnetii* bacteria (green) within two epithelial cells in a lactiferous lobule by fluorescence in situ hybridization (FISH). Four different fluorescein labeled oligonucleotide probes specifically targeting 16 S ribosomal RNA of *C. burnetii* were used. Bar = 20 μm

*C. burnetii* bacteria were identified in the mammary gland tissue by FISH (Fig. 6). In both mammary gland tissues, a few spots with 1–3 cells contained cytoplasmic granular fluorescence (Fig. 6). The fluorescing cells were either part of the epithelial lining or were found free in the lactiferous lobules.

## Discussion

In the present study, *C. burnetii* was diagnosed as the abortifacient pathogen in a dairy goat herd, and a vaccination program was implemented to control the disease and prevent excretion of the pathogen. Nevertheless, individually vaccinated goats shed *C. burnetii* sporadically over a period of approximately two years, which might have resulted in positive BTM samples, and this is in line with previous studies (Bauer et al. 2022; Boarbi et al. 2014; van den Brom et al. 2013). Therefore, vaccination of *C. burnetii* infected goats may not completely prevent *C. burnetii* excretion in milk, but may reduce it (Hogerwerf et al. 2011). In previous experimental studies, *C. burnetii* DNA was detected in mammary gland tissue from both udder halves and in the supramammary lymph nodes (Roest et al. 2012, 2020; Sánchez et al. 2006). In natural *C. burnetii* infected and vaccinated dairy goats, *C. burnetii* DNA was also identified in mammary gland tissue, but their supramammary lymph nodes tested negative, which is consistent with our findings. Viable *C. burnetii* microorganisms have not yet been detected in mammary gland tissue from ruminants. Although, *C. burnetii* showed in vitro a high replication rate in epithelial cells from bovine udder compared to epithelial cells from lung and placenta (Sobotta et al. 2017). The tropism of *C. burnetii* to mammary gland tissue seems to provide the basis for excretion in milk. Infection of the mammary gland appears to occur regardless of the pregnancy status of the goat (Roest et al. 2012, 2020). Moreover, the risk of *C. burnetii* transmission among goats through milk during milking activity remains uncertain. An experimental infection of dairy cows through the teat canal resulted in a five-day bacteremia and *C. burnetii* milk shedding for 63 days (Williams 1991). In contrast, dipping cattle teats into *C. burnetii*-contaminated milk for 10–19 weeks failed to establish an infection (Williams 1991). Taken together, the reasons and associated risks of chronic milk shedders in ruminants needs further targeted investigations.

An important question that arose in our study is why *C. burnetii* colonizes the udder in addition to its tropism for trophoblasts. For example, similar low/reduced oxygen levels in trophoblasts and the udder may play a role for the bacterial tissue tropism. So one factor might be the oxygen content in the udder tissue. Indeed, in goats, O_2_ uptake in the udder increases during late gestation and peaks during early lactation (Davis et al. 1979). The increasing oxygen consumption is thought to lead to localized chronic hypoxia (Zhao 2014). Hypoxia in mammary gland tissue and the accumulation of hypoxia-inducible factor (HIF)-1α could impair bacterial clearance and allow recurrent or chronic *C. burnetii* manifestation to occur (Hayek et al. 2022). Hypoxic conditions are found in inflamed and infected tissues (Jantsch and Schödel 2015), and chronic subclinical mastitis increases the likelihood of *C. burnetii* shedding in milk in cattle (Barlow et al. 2008). Our hypothesis is supported by the detection of *C. burnetii* in arteriosclerotic plaques in humans, which are also considered hypoxic (Hagenaars et al. 2014; Jantsch and Schödel 2015). Furthermore, *C. burnetii* was successfully cultivated from a diseased human heart valve under hypoxic conditions (Boden et al. 2015). Finally, the conditions that favor the colonization of *C. burnetii* in body tissues, including the mammary gland, need more elucidation in the future.

No specific pathogens were detected in routine bacteriological milk examination. Nevertheless, a mild, predominantly interstitial lymphoplasmacytic inflammation was found histopathologically in the mammary gland. Extensive lymphocytic infiltration was observed in the mammary gland from one goat after experimental infection with *C. burnetii* (Sánchez et al. 2006). In addition, CAEV also causes interstitial lymphocytic inflammation in the goat udder (Zink and Johnson 1994), but antibodies against CAEV were not detected in the goat by ELISA. Moreover, focal granulomatous inflammation was found in the mammary gland tissue of goat #67455. Granulomatous inflammation can be caused by various infectious and non-infectious reasons (Shah et al. 2017). In the present case granulomatous inflammation was always localized around corpora amylacea and was most likely interpreted as a foreign body reaction. Infectious causes of granuloma formation in goat udders are for example, *Mycobacterium caprae* and yeasts, and affected goats show severe clinical signs of mastitis (Ahmed et al. 2020; Singh et al. 1994), which were not present in the current case. Acute *C. burnetii* infection also leads to the formation of granulomas in infected human organs, and a lipid vacuole forms the center of these *C. burnetii*-specific granulomas (Raoult et al. 2005). However, granulomas have rarely been observed in chronic Q fever patients, and it is suggested that the absence of typical granulomas is due to the lack of a T-cell immune response (Maurin and Raoult 1999). Finally, *C. burnetii* did not induce the upregulation of pro-inflammatory cytokines such as IL-1β, IL-6 and TNF-α, in bovine udder epithelia (Sobotta et al. 2017). Therefore, the histopathological findings revealed in goat #67455 can not be related to the presence of *C. burnetii*. The minor clinical impact of *C. burnetii* in the mammary gland is supported by the SCC value (< 300 × 10^3^ cells/ml) of the bilateral milk specimens on the day of euthanasia. There is no legal limit for SCC in goats in the European Union. The SCC value varies widely between 270 and 2,000 × 10^3^ cells/ml in goats without intramammary infection and also depends on physiological factors (Haenlein 2002; Paape et al. 2001). Therefore, we assume that the *C. burnetii* infection had no impact on the SCC of the present goat. In contrast, the SCC in cattle seems to be negatively influenced by *C. burnetii* (Barlow et al. 2008).

The BTM samples remain *C. burnetii* DNA negative after the removal of goat #67455. This does not rule out the excretion of the pathogen by others goats, as shown in Fig. 2. However, goat #67455 excreted the largest amount of *C. burnetii* DNA during sampling in November 2020 and March 2021, and BTM also tested positive on the same sample dates (Fig. 1). In contrast, no or only a small amount of *C. burnetii* DNA was detected in individual milk samples from goat #67455 (January and July 2020), but the BTM tested negative on the same dates. Therefore, the detection of *C. burnetii* in BTM appears to depend on the amount of pathogen excreted and the dilution effect of BTM. Studies comparing *C. burnetii* outcomes among individual milk shedders and BTM are rare. In dairy cows, a sensitivity of 82% (95% CI 69–95%) and a specificity of 70% (95% CI 59–81%) were estimated for BTM samples (Muskens et al. 2011). Similar results were reported by van den Brom and colleagues (2012) who evaluated a PCR assay for the detection of *C. burnetii* in BTM samples from goats. Finally, a single BTM sample can give a false-negative result, therefore repeated testing is required to confirm herd status.

Nowadays, BTM samples are commonly used to identify dairy goat herds infected with *C. burnetii* (Jansen et al. 2021; Jodełko et al. 2021; Khalili et al. 2015). Viable *C. burnetii* has been detected in raw milk from cattle (Loftis et al. 2010) and in raw milk cheese from sheep for up to 8 months (Barandika et al. 2019). This poses a risk for humans to become infected with Q fever through consumption of raw milk, which is becoming increasingly popular. Although seroconversion does occur after consumption of contaminated raw milk, acute Q fever cases are rare and appear to depend on the amount of milk consumed (Signs et al. 2012). Recent data suggest that the pasteurization temperature, according to the Codex Alimentarius, can be lowered by 1–2˚C to inactivate *C. burnetii* in raw milk, and this should be applied to prevent alimentary transmission (Wittwer et al. 2022).

The authors are aware of the limitation of the study. Only one goat was studied, but this is the first time that the presence of *C. burnetii* antigen and bacterial cells in mammary gland tissue has been demonstrated with IHC and FISH, respectively. Mammary gland tissue manifestation was not achieved by experimental conditions. Therefore, the findings from our field study are a further contribution to confirming the occurrence of chronic *C. burnetii* excretion in milk in dairy animals, although the reason for the potential manifestation of *C. burnetii* in the mammary gland tissue remains uncertain. Furthermore, a hypothesis on the possible influence of hypoxia in the udder on Coxiella colonization/replication has been proposed, which requires more intensive investigations under controlled conditions.

This field study demonstrates for the first time the presence of *C. burnetii* antigen and bacterial cells in mammary gland tissue from a naturally infected dairy goat using IHC and FISH, respectively. Moreover, the examined goat shed *C. burnetii* through milk on three out of four sampling dates and during a four-week examination period, which might be caused by the presence of *C. burnetii* in the mammary gland tissue. Persistent *C. burnetii* shedders should be removed from the herd to minimize the risk of *C. burnetii* transmission among animals, although the role of contaminated milk as an infectious source remains unclear. The growing popularity of purchasing raw milk directly from farms and consuming it without prior heating (EFSA Panel on Biological Hazards 2015; Sobotta et al. 2022) might pose a risk for acquiring Q fever. Although the digestive route might not pose a major public health threat, it could potentially play a role in the transmission of *C. burnetii* (Eldin et al. 2017). Therefore, heat treatment of raw milk from *C. burnetii*-infected herds is recommended (BfR 2010). This recommendation should also be applied to milk from goats that are both *C. burnetii*-positive and subsequently vaccinated, as vaccination appears unable to prevent *C. burnetii* shedding (Bauer et al. 2022).

## Data Availability

The datasets generated during and/or analyzed during the current study are available from the corresponding author on reasonable request.
